# Thanksgiving Dinner

**Published:** 1858-01

**Authors:** 


### THANKSGIVING DINNER.

Mr. Editor—Your monthly budget of counsels for the preservation of health is absolutely beyond price. But what if you had a kind of "*Confessional*" for the record of the grievous sins which your readers and patients commit against their bodies. Would not this serve a good purpose? The advantages of such a thing are obvious; the great difficulty would be, to get any body to publish the history of their follies.

De Quincy has given us the "Confessions of an Opium-Eater." Who will write the Confessions of a Dyspeptic? What a book it would be—a new feature in morbid anatomy—a curiosity-shop of jellies, sauces, and surfeits, mixed up with aches and vapors, and lethargies, fits of melancholy and anger, turns of the "dumps" with interludes of sharp reproaches from the tongue of an outraged conscience. The man who has the courage, the magnanimity and the descriptive power equal to production of such a book, may earn immortality in the attempt, besides conferring an inestimable boon on his species.

But about that "Thanksgiving Dinner." It occured on the 26th of November last, and besides being Saint Bartholemew's day to the turkeys, it was a day of massacre to my peace both of body and mind.

My only safety is in habitual abstemiousness: I knew this well enough, and knew likewise that a man might about as well try to keep Lent during the Christmas holidays as essay to be temperate at a Thanksgiving dinner. Eight courses were required to finish the meal, about half an hour to each course. The idea of spending four hours in eating a *moderate* repast! All my resolution was summoned up to be temperate. I discarded utterly pies, puddings, and jellies, and gave a contemptuous nod to all tempting *entrés;* but after all, what was the result? A fit of indigestion, which lasted about a week—such bitter recollections, such smitings of conscience, such fastings and watchings to get the digestive apparatus into joint again! The conclusion is, that it didn't pay. Better by far that a man who is trying to fight against chronic dyspepsia should say: "No, thank ye," to hospitality of this epicurean sort, and eat his frugal meal of bread and meat at home with a thankful heart and a quiet conscience. Will you give your editorial endorsement to this? If you do, it implies a good sound professional scolding, which I shall consider personal to myself, an *argumentum ad hominem*, which I confess I richly deserve. C.

